# Validating the CG-STYLE Measure in Family Caregivers of Individuals Living With Dementia

**DOI:** 10.1093/geroni/igaf122.1571

**Published:** 2025-12-31

**Authors:** Amanda Leggett, Jonathan Troost, Jennifer Miner, Sophia Tsuker, Jin-Shei Lai, Noelle Carlozzi

**Affiliations:** Wayne State University, Detroit, Michigan, United States; University of Michigan, Ann Arbor, Michigan, United States; University of Michigan, Ann Arbor, Michigan, United States; Wayne State University, Detroit, Michigan, United States; Northwestern University, Evanston, Illinois, United States; University of Michigan, Ann Arbor, Michigan, United States

## Abstract

Family caregivers for individuals with dementia provide care based on unique caregiving styles. Using mixed-methods approaches, we developed a measure representing five caregiving style domains: understanding, adaptability, emotional expression (positive, negative), orientation to self-or-other, and behavioral management strategies (adaptive, maladaptive). We present data supporting the reliability and validity of this new Caregiving Style (CG-STYLE) measure. The sample included 209 family/friend caregivers for a community-dwelling individual living with dementia. Participants completed the CG-STYLE measure and comparator measures. We explored floor and ceiling effects (criterion ≤20%), convergent validity (correlations between similar domains should be moderate: 0.36 - 0.67 or high: 0.68 - 0.89), discriminant validity (correlations between dissimilar domains should be small: ≤ 0.3), and known groups validity (there should be significant differences between groups known to be different; determined by high versus low scores [median split] on caregiver readiness, competence, criticism, and active management). The CG-STYLE domains were devoid of floor or ceiling effects apart from a slight ceiling effect (30.3%) for maladaptive behavioral management strategies. There were moderate to high correlations between the CG-STYLE domains and known comparators supporting convergent validity, and negligible to small correlations between the CG-STYLE domains and an unrelated construct (i.e., pain intensity) supporting discriminant validity. There were significant group differences for all CG-STYLE domains (Cohen’s d’s range from 0.38 to 1.68). Findings support the preliminary reliability and validity of the CG-STYLE measure suggesting its utility in research, community, and clinical practice to target and tailor supports to caregiver’s unique caregiving styles.

